# Common Variations in *BMP4* Confer Genetic Susceptibility to Sporadic Congenital Heart Disease in a Han Chinese Population

**DOI:** 10.1007/s00246-014-0951-1

**Published:** 2014-07-15

**Authors:** Bo Qian, Ran Mo, Min Da, Wei Peng, Yuanli Hu, Xuming Mo

**Affiliations:** 1 Department of Cardiothoracic Surgery, The Affiliated Children’s Hospital of Nanjing Medical University, No. 72 Guangzhou Road, Nanjing, 210008 China; 2 Medical School of Nanjing University, Nanjing, China

**Keywords:** Congenital heart disease, Single nucleotide polymorphism, Bone morphogenetic protein 4

## Abstract

Congenital heart disease (CHD) is the most common birth defect in humans. The genetic causes of sporadic CHD remain largely unknown. Bone morphogenetic protein 4 (BMP4), a member of the transforming growth factor-β (TGF-β) family, is required for normal heart development. Loss of *BMP4* gene expression in mice is associated with septal defects, defective endocardial cushion remodeling, and abnormal semilunar valve formation. This study evaluated the contribution of single nucleotide polymorphisms (SNPs) in *BMP4* to CHD susceptibility in a case–control study of 575 patients with CHD and 844 non-CHD control subjects in a Chinese population. The *BMP4* SNP rs762642 was associated with CHD in an additive model (odds ratio [OR]_add_ 1.22; 95 % confidence interval [CI] 1.04–1.43; *P*_add_ = 0.02). Stratified analysis by CHD subtypes showed a significant association only between rs762642 and atrial septal defect (OR_add_ 1.33; 95 % CI 1.04–1.72; *P*_add _= 0.03) in the additive model. This study was the first to indicate that a common variant of *BMP4* may contribute to susceptibility to sporadic CHD in a Chinese population.

## Introduction

Congenital heart disease (CHD) is a leading cause of perinatal mortality, with an incidence of approximately 6–8 in 1,000 live births [6]. For classification of CHD, three broad categories have been defined: the more common septation defects, cyanotic heart disease, and left-sided obstruction defects [1]. However, the proportion of each category varies greatly among geographic regions.

The origin of CHD is complex and involves both environmental and genetic factors. Recognizable chromosomal variants account for 13 % of all CHD patients, whereas most CHD occurs sporadically [16]. The mechanisms regulating early heart development and morphogenesis have been studied extensively in recent years [15]. Given that dysregulation of heart development is at the root of the disease, a clear picture of how the heart develops is crucial to our understanding of the genesis of CHD [1].

Cardiogenesis involves a complex series of coordinated tissue morphogenetic events. During cardiac looping, the heart tube elongates by the addition of progenitor cells from the second heart field to generate the four chambers of the mature heart. Endocardial cells that overlie the cardiac cushions subsequently undergo epithelial-to-mesenchymal transformation (EMT) and ultimately contribute to all the valves and septa of the mature heart [7]. Cardiac neural crest cells also migrate into the developing heart and promote normal cardiac development [9].

A complex network of signaling inputs and transcriptional regulators has recently been shown to control CHD development. These include fibroblast growth factor (FGF), bone morphogenetic protein (BMP), Notch, and the transcription factor Islet-1 (ISL1) [10]. Minor imbalances in this network during cardiovascular development can result in profound CHDs, including septal defects, which represent the most common CHD [1].

The BMP subclass of transforming growth factor (TGF-β) signaling molecules includes BMP4. Findings have shown that the BMP4 signaling molecule is expressed in the ventral splanchnic and branchial-arch mesoderm and in the myocardium of the outflow tract in mice, suggesting that it may play an important role in cardiac development [12]. Furthermore, BMP4 has been reported to regulate EMT and cardiac neural crest migration together with Notch signals as a downstream target of FGF8 [5]. Loss of *BMP4* gene expression in mice resulted in septal defects, defective endocardial cushion remodeling, and abnormal semilunar valve formation [14].

Recent studies have suggested that BMP4 directly regulates a micro RNA (miRNA)-mediated effector mechanism, which in turn downregulates cardiac progenitor genes and enhances myocardial differentiation by reducing gene expression of *ISL1* and T-box transcription factor 1 (*TBX1*), which are vital transcription factors for normal cardiac development [13, 20].

We tested the hypothesis that single nucleotide polymorphisms (SNPs) in the *BMP4* gene may be associated with CHD risk by performing genotyping analyses of *BMP4* polymorphisms in a case–control study of 575 CHD patients and 844 control subjects in an ethnic Han Chinese population.

## Materials and Methods

### Study Population

The study was approved by the institutional review board of Nanjing Children’s Hospital Affiliated with Nanjing Medical University, Nanjing, China. In brief, this hospital-based case–control study included 575 confirmed CHD patients and 844 non-CHD control subjects. All the CHD patients had either atrial septal defects (ASDs) or ventricular septal defects (VSDs) (the most common CHDs) diagnosed by ultrasound and confirmed in surgical operations.

The CHD patients were recruited consecutively from the Affiliated Nanjing Children’s Hospital of Nanjing Medical University, Nanjing, China, between July 2011 and August 2012. Structural malformations involving another organ system and known chromosomal abnormalities were excluded in all patients. Additional exclusion criteria specified a positive family history of CHD in a first-degree relative (parents, siblings, and children), maternal diabetes mellitus, phenylketonuria, maternal teratogen exposures (e.g., pesticides and organic solvents), and maternal therapeutic drug exposure during the intrauterine period.

The control subjects were non-CHD outpatients recruited from the same hospital during the same period, mostly with a diagnosis of trauma or infection. Control subjects with congenital anomalies were excluded from the study. All the subjects were genetically unrelated ethnic Han Chinese. Informed consent was obtained directly or from the patient’s parents.

The subjects, their parents, or both were interviewed personally by trained interviewers using a structured questionnaire. After the interview, 4 mL of venous blood was collected from each subject.

### SNP Selection and Genotyping

The *BMP4* gene is located on chromosome 14q22.2. We used the public HapMap SNP database (phase 2 + 3 February 2009 on NCBI B36 assembly, dbSNP b126) to search for SNPs located in each gene or in the 2-kb upstream region of the gene with a minor allele frequency (MAF) of 0.05 or higher in the Han Chinese Han population (CHB). We then performed linkage disequilibrium analysis and defined three SNPs (rs17563, rs2071047, rs762642) as the tagging SNPs based on an *r* ^2^ threshold of 0.8 by Haploview software.

### DNA Extraction and Genotyping

Genomic DNA was isolated from leukocyte pellets of venous blood by proteinase K digestion followed by phenol-chloroform extraction as described previously [25]. The SNPs were genotyped using the TaqMan assay on the ABI PRISM 7900 HT platform (Applied Biosystems, Inc., Foster City, California). Primers and probes were available on request (rs2761887-F: TCAGAGCAAGGCCAAGACATC; R: GACTTGCCCAAAGCCATACAG; P-C: FAM-TGCCATCTTTTCCATC-MGB; P-A: HEX-TGCCATATTTTCC-MGB. rs17563, F: TGCTTTTCGTTTCCTCTTTAACCT; R: ACGGTGGAAGCCCCTTTC; P-T: FAM-CGAGGTGATCTCC-MGB; P-C: HEX-TGAGAACGAGGCGAT-MGB. rs2071047, F: CACGTCTGAGTGCTGTCATTCC; R: TGCTTACCTCCAAGAATTTGCA; P-T: FAM-AGCTGCCGCTGAA-MGB; P-C: HEX-CTGCCGCCGAACT-MGB. rs762642, F: TGGCGCTGAAAGACCTTTAAA; R :CCAAGGGCTTCTCTTGTTTCTG; P-G: FAM-CTGGGAGAGTCTCACCT-MGB; P-T: HEX-TGGGAGATTCTCACC-MGB).

Genotyping was performed blinded to the case or control status. Two blank (water) wells in each 384-well plate were used for quality control, and at least 10 % of the samples were randomly selected for repeat analysis, which showed 100 % concordance. All three SNPs were successfully genotyped with call rates of 95 % or higher.

### Statistical Analyses

Differences in the distributions of genotype frequencies of the polymorphisms between the cases and the control subjects were evaluated using chi-square or Student’s *t* tests. The Hardy-Weinberg equilibrium was tested by a goodness-of-fit chi-square test to compare the observed with the expected genotype frequencies among the control subjects. Associations between genotype and risk of CHD were estimated by odds ratios (ORs) and 95 % confidence intervals (CIs) from logistic regression analyses. All statistical analyses were performed using Statistical Analysis System software (v.9.1.3e; SAS Institute, Cary, North Carolina).

## Results

The characteristics of the CHD patients and the control subjects are summarized in Table 1. The patients and the control subjects did not differ significantly in terms of age or sex. Of the 575 patients with CHD, 165 (28.7 %) had VSDs and 410 (71.3 %) had ASDs. The genotype distributions of these SNPs in the CHD patients and control subjects are shown in Table 2.

**Table 1:** Demographic and Selected variables among patients with congenital heart disease (CHD) and control subjects

	CHD *n* (%)	Controls *n* (%)	*P* Value
CHD classification	575	―	<0.001
VSD	410 (71.3)	―	
ASD	165 (28.7)	―	
Sex	575	844	0.52
Male	296 (51.5)	420 (49.8)	
Female	279 (48.5)	424 (50.2)	
Mean age (years)	6.86 ± 4.34	6.59 ± 4.81	0.28

*VSD* ventricular septal defect, *ASD* atrial septal defect

**Table 2:** Associations between *BMP4* SNPs and risk of congenital heart disease (CHD) in a Chinese population

SNPs	Genotype	CHD^a^	Controls^a^	MAF		OR_dom_ (95 % CI)^b^	OR_rec_ (95 % CI)^c^	OR_het_ (95 % CI)^d^	OR_homo_ (95 % CI)^e^	OR_add_ (95 % CI)^f^	*P*_add_^g^
				CHD	Controls						
rs2761887	A/C^h^	128/302/145	216/411/217	0.49	0.50	1.02 (0.80–1.30)	0.83 (0.64–1.06)	1.10 (0.85–1.42)	0.88 (0.65–1.19)	0.94 (0.81–1.09)	0.42
rs17563	A/G^h^	37/244/294	57/356/431	0.28	0.28	1.00 (0.81–1.23)	0.95 (0.62–1.46)	1.01 (0.81–1.25)	0.95 (0.61–1.48)	0.99 (0.83–1.18)	0.91
rs2071047	G/A^h^	89/284/202	119/402/323	0.40	0.38	1.15 (0.92–1.43)	1.12 (0.83–1.50)	1.13 (0.90–1.43)	1.20 (0.86–1.66)	1.10 (0.94–1.29)	0.22
rs762642	A/C^h^	76/276/223	78/400/366	0.37	0.33	1.21 (0.97–1.50)	1.50 (1.07–2.09)	1.13 (0.90–1.42)	1.60 (1.12–2.29)	1.22 (1.04–1.43)	0.02

*BMP4* bone morphogenetic protein 4, *SNP* single nucleotide polymorphism, *MAF* minor allele frequency, *OR* odds ratio, *CI* confidence interval

^a^Variant homozygote/heterozygote/wild-type homozygote

^b^OR_dom_, wild-type homozygote vs heterozygote and variant homozygote

^c^OR_rec_, wild-type homozygote and heterozygote vs variant homozygote

^d^OR_het_, heterozygote vs wild-type homozygote

^e^OR_homo_, variant homozygote vs wild-type homozygote

^f^OR_add_, calculated by the additive model

^g^ *P*_add_, calculated by the additive model

^h^Major/minor alleles

The observed genotype frequencies for all four variants were in Hardy-Weinberg equilibrium among the control subjects. Logistic regression analysis showed that the *BMP4* rs762642 variant genotype was associated with a significantly increased risk of CHD according to the additive model (OR, 1.22; 95 % CI, 1.04–1.43). We also found significant associations in the recessive model (OR, 1.50; 95 % CI, 1.07–2.09) and the co-dominant model (variant homozygote vs wild-type homozygote: OR, 1.60; 95 % CI, 1.12–2.29). Stratified analyses of these three SNPs are summarized in Table 3.

**Table 3:** Stratified analysis by congenital heart disease (CHD) subtype

SNPs	Subtype	CHD^a^	Controls^a^	MAF		OR_dom_ (95 % CI)^b^	OR_rec_ (95 % CI)^c^	OR_het_ (95 % CI)^d^	OR_homo_ (95 % CI)^e^	OR_add_ (95 % CI)^f^	*P*_add_^g^	*P* ^h^
				CHD	Controls							
rs2761887	ASD	29/91/45	216/411/217	0.45	0.50	0.92 (0.63–1.34)	0.62 (0.40–0.95)	1.06 (0.72–1.58)	0.64 (0.39–1.06)	0.82 (0.65–1.04)	0.10	0.203
A/C^a^	VSD	99/211/100	216/411/217	0.50	0.50	0.92 (0.70–1.21)	1.07 (0.81–1.40)	0.88 (0.67–1.19)	0.97 (0.70–1.40)	0.99 (0.84–1.18)	0.93	
rs17563	ASD	8/77/80	57/356/431	0.28	0.28	1.11 (0.79–1.55)	0.70 (0.33–1.50)	1.17 (0.83–1.64)	0.76 (0.35–1.65)	1.02 (0.78–1.34)	0.90	0.813
A/G^a^	VSD	29/167/214	57/356/431	0.27	0.28	0.96 (0.75–1.21)	1.05 (0.66–1.67)	0.94 (0.74–1.21)	1.03 (0.64–1.65)	0.98 (0.81–1.19)	0.83	
rs2071047	ASD	28/81/56	119/402/323	0.42	0.38	1.21 (0.85–1.71)	0.25 (0.79–0.95)	1.16 (0.80–1.68)	1.36 (0.82–2.24)	1.16 (0.91–1.48)	0.22	0.637
G/A^a^	VSD	61/203/146	119/402/323	0.40	0.38	1.12 (0.88–1.43)	1.07 (0.76–1.49)	1.12 (0.86–1.45)	1.13 (0.79–1.63)	1.08 (0.91–1.28)	0.40	
rs762642	ASD	23/83/59	78/400/366	0.39	0.33	1.38 (0.97–1.95)	1.59 (0.97–2.62)	1.29 (0.90–1.85)	1.83 (1.06–3.14)	1.33 (1.04–1.72)	0.03	0.450
A/C^a^	VSD	53/193/164	78/400/366	0.36	0.33	1.15 (0.90–1.46)	1.46 (1.01–2.11)	1.08 (0.84–1.39)	1.52 (1.02–2.25)	1.18 (0.98–1.41)	0.07	

*SNP* single nucleotide polymorphism, *MAF* minor allele frequency, *OR* odds ratio, *CI* confidence interval, *ASD* atrial septal defect, *VSD* ventricular septal defect

^a^Variant homozygote/heterozygote/wild-type homozygote

^b^OR_dom_, wild-type homozygote versus heterozygote and variant homozygote

^c^OR_rec_, wild-type homozygote and heterozygote vs variant homozygote

^d^OR_het_, heterozygote versus wild-type homozygote

^e^OR_homo_, variant homozygote vs wild-type homozygote

^f^OR_add_, calculated by the additive model

^g^ *P*_add_, calculated by the additive model

^h^ *P*, heterogeneity test between groups

The stratified analysis by CHD subtype showed only a significant association between rs762642 and ASD (OR_add_ 1.33; 95 % CI 1.04–1.72; *P*_add _= 0.03) in the additive model. Comparison of variant homozygotes with wild-type homozygotes in the co-dominant model showed that rs762642 was significantly associated with risks of ASD (OR 1.83; 95 % CI 1.06–3.14) and VSD (OR 1.52; 95 % CI 1.02–2.25). There was no significant heterogeneity between the subgroups for all the SNPs.

## Discussion

The results of this study demonstrated a significantly increased risk of CHD associated with the *BMP4* (rs762642) polymorphism, suggesting that this polymorphism may contribute to susceptibility to CHD. Stratified analysis by CHD subtype indicated that the rs762642 polymorphism conferred susceptibility to ASD. To the best of our knowledge, this is the first study to evaluate the association between *BMP4* polymorphisms and CHD risk.

The BMP proteins are members of the TGF-β signaling family, which bind to heterotetrameric receptor complexes and exert their effects via receptor-mediated activation of Smad transcription factors [3]. Activated Smads then translocate to the nucleus to alter the expression of downstream transcriptional targets and regulate many aspects of skeletal, cardiovascular, craniofacial, and limb development [22].

Findings show that BMPs play diverse roles in valve development and cardiac septation. Myocardial BMP2 activates the expression of hyaluronic acid synthetase 2 to produce the cushion extracellular matrix required for EMT [17], whereas the BMP2 and BMP4 receptor BMPR2 plays various spatial roles. Mice with hypomorphic alleles of *BMPR2* exhibit interruption of the aortic arch, persistent truncus arteriosus, and missing semilunar valves [2].

The *BMP4* gene is located on chromosome 14q22.2. It comprises four exons and three introns, among which exons 3 and 4 encode the protein (http://genome.ucsc.edu/cgi-bin). The BMP4 signaling molecule is expressed in the ventral splanchnic- and branchial-arch mesoderm and in the cardiac outflow tract myocardium [12]. Endocardial cells in defined areas of the outflow tract undergo EMT and invade the intervening space to form the endocardial cushions, induced by BMP4. In addition, BMP4 also induces the cardiac neural crest to invade the forming aortopulmonary septum and outflow tract cushions [8].

High et al. [5] reported that Notch signals affected EMT and induced the neural crest by regulating FGF8, which functioned within the second heart field to regulate downstream cascades including BMP4, suggesting that multiple signaling inputs participate in cardiac development [5]. Inactivation of *BMP4* within the myocardium results in neonatal lethality as a result of severe defects in septation and valve diseases such as ASD and VSD.

Recent studies have suggested that BMP4 may directly regulate an miRNA-mediated effector mechanism that downregulates cardiac progenitor genes and enhances myocardial differentiation by reducing expression of ISL1 and TBX1 [20]. The transcription factor ISL1 marks cardiac progenitor cells and generates diverse multipotent cardiovascular cell lineages, directing complex cardiac morphogenesis. During cardiac development, ISL1 clearly acts as an upstream transcription factor capable of activating various downstream targets.

The dosage-sensitive regulator TBX1 plays a critical role in heart development. Mutations in the *TBX1* gene have been associated with major phenotypes in 22q11 deletion syndromes such as DiGeorge syndrome, conotruncal anomaly face syndrome, and velo-cardiofacial syndrome. These mutations also feature in thymic hypoplasia, parathyroid hypoplasia, and cardiac defects such as VSD, tetralogy of Fallot, and double-outflow right ventricle [18]. In heart development, TBX1 interacts with signaling molecules and transcription factors to regulate its downstream target genes [21]. Normal cardiac development involves a network of complex interconnected signaling pathways and transcription factors designed to balance proliferation, migration, and subsequent differentiation and to minimize errors [4, 23]. As a vital part of this complex network, BMP4 regulates cardiac normal development, which includes multiple signaling inputs and transcription factors.

Lin et al. [11] recently demonstrated significant differences in the genotype and allele distributions of the 538T/C (rs17563) polymorphism of the *BMP4* gene among patients with nonsyndromic cleft lip with or without cleft palate (NSCLP) and control subjects in Chinese children. The 538 C allele carriers had a significantly greater risk of NSCLP than the noncarriers.

In a Chilean study, the *BMP4* (rs762642) G/T polymorphism was significantly associated with NSCLP cases [19]. However, no previous studies have addressed the association between *BMP4* polymorphisms and CHD susceptibility.

The *BMP4* promoter region contains the elements that control the initiation and regulation of transcription. The SNP rs762642 is located within the first intron of *BMP4* near the promoter and an enhancer region. Previous research showed functional SNPs in the noncoding region of folate-related genes in birth defects. We therefore hypothesized that the *BMP4* rs762642 polymorphism could have a functional effect on BMP4 expression or protein activity, causing an imbalance in the network responsible for regulating normal cardiac development [24]. The *BMP4* rs2071047 polymorphism is located in the intron region, but we found no significant association between this SNP and CHD risk.

Our study had certain limitations. It analyzed a hospital-based population and may therefore have been subject to selection bias of subjects associated with a particular genotype. The control patients were non-CHD outpatients from the same geographic area. Further functional studies are needed to validate our current findings. In addition, this study was conducted with a Han Chinese population, and the results should therefore be extrapolated to other ethnic groups with caution.

In conclusion, the results of this study indicate that the *BMP4* rs762642 C polymorphism may confer susceptibility to sporadic CHD. Stratified analysis by CHD subtype suggests that the rs762642 polymorphism contributes to susceptibilities to both ASD and VSD. However, the sample size was moderate, so the statistical power of the study was limited. Larger population-based, prospective studies are needed to clarify the impact of *BMP4* polymorphisms on CHD susceptibility. In addition to providing new insights into the variety of CHD phenotypes, our observations also may provide the basis for a more integrated understanding concerning the molecular basis of human CHD and will increase our appreciation of interactions among regulatory pathways and of how the heart can compensate for imbalances.
